# Thermal structure of the Venusian atmosphere from the sub-cloud region to the mesosphere as observed by radio occultation

**DOI:** 10.1038/s41598-020-59278-8

**Published:** 2020-02-26

**Authors:** Hiroki Ando, Takeshi Imamura, Silvia Tellmann, Martin Pätzold, Bernd Häusler, Norihiko Sugimoto, Masahiro Takagi, Hideo Sagawa, Sanjay Limaye, Yoshihisa Matsuda, Raj Kumar Choudhary, Maria Antonita

**Affiliations:** 1 Faculty of Science, Kyoto Sangyo University, Kyoto, Japan; 2 Department of Complexity Science and Engineering, The University of Tokyo, Chiba, Japan; 3 Abteilung Planetenforschung, Rheinisches Institut für Umweltforschung, Universität zu Köln, Köln, Germany; 4 Institut für Raumfahrttechnik und Weltraumnutzung, Universität der Bundeswehr München, Neubiberg, Germany; 5 Research and Education Center for Natural Sciences, Department of Physics, Keio University, Kanagawa, Japan; 6 University of Wisconsin, Wisconsin, USA; 7 Department of Astronomy and Earth Sciences, Tokyo Gakugei University, Tokyo, Japan; 8 Vikram Sarabhai Space Center, Space Physics Laboratory, Thiruvananthapuram, India; 9 Indian Space Research Organization, Bengaluru, India

**Keywords:** Atmospheric dynamics, Atmospheric dynamics

## Abstract

We present distributions of the zonal-mean temperature and static stability in the Venusian atmosphere obtained from Venus Express and Akatsuki radio occultation profiles penetrating down to an altitude of 40 km. At latitudes equatorward of 75°, static stability derived from the observed temperature profiles is consistent with previous *in-situ* measurements in that there is a low-stability layer at altitudes of 50–58 km and highly and moderately stratified layers above 58 km and below 50 km, respectively. Meanwhile, at latitudes poleward of 75°, a low-stability layer extends down to 42 km, which has been unreported in analyses of previous measurements. The deep low-stability layer in the polar region cannot be explained by vertical convection in the middle/lower cloud layer, and the present result thus introduces new constraints on the dynamics of the sub-cloud atmosphere. The Venusian atmosphere is in striking contrast to the Earth’s troposphere, which generally has a deeper low-stability layer at low latitudes than at mid- and high latitudes.

## Introduction

The dynamics of the Venusian atmosphere remain unclear because Venus is completely globally covered by thick clouds at altitudes of 48–70 km. The thermal structure of the Venusian atmosphere across this cloud layer is important in terms of understanding aspects of the general circulation, such as the mean meridional circulation and baroclinic instability waves, which contribute to meridional heat transport and atmospheric super-rotation. *In-situ* measurements were made by entry probes around the equator and 60°N as part of the Venera and Pioneer Venus missions, revealing a low-stability (weakly or almost neutrally stratified) layer at an altitude of approximately 50–55 km, a highly stratified layer above 55 km and a moderately stratified layer below 50 km^1^. Radio occultation measurements, one of the most useful methods of obtaining vertical temperature profiles, were made as part of the National Aeronautics and Space Administration’s (NASA’s) Pioneer Venus mission and the European Space Agency’s (ESA’s) Venus Express mission^2–4^ to obtain latitude–height distributions of temperature. These observations revealed that a cold latitudinal band called a “cold collar”, which is thought to be formed by dynamics^5^ and/or the latitudinal cloud structure^6^, is located at roughly 65° latitude near the cloud top (at nearly 65 km altitude) and surrounds a warm polar region and that temperature increases with latitude above 65 km and decreases with latitude below 60 km.

Furthermore, static stability profiles obtained from the Venus Express radio occultation measurements showed that the low-stability layer in the cloud layer is deeper at high latitudes than at low and mid-latitudes^4^. However, no *in-situ* measurements have been made at latitudes poleward of 60°, and the thermal structure below the cloud layer at high latitudes thus remains unknown.

**Figure 1:**
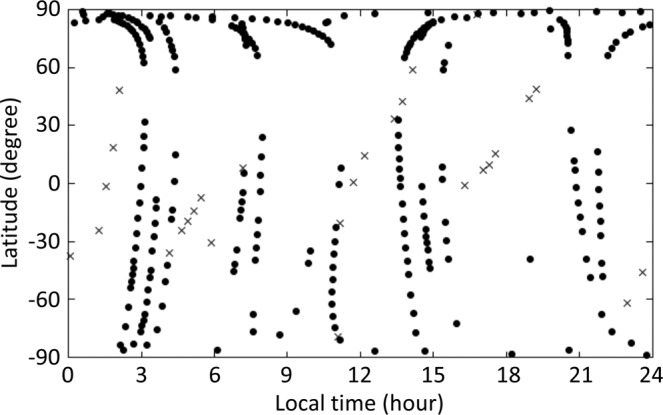
Local time–latitude distribution of radio occultation measurements made on Venus Express (dots) and Akatsuki (crosses) missions. An observation point is defined as the tangential point of the straight line connecting the spacecraft and tracking station at the moment that the spacecraft is occulted by the 50-km-altitude surface.

The present study investigated the thermal structure of the Venusian atmosphere using temperature profiles obtained by ESA’s Venus Express and Japan Aerospace eXploration Agency’s (JAXA’s) Akatsuki radio occultation measurements to highlight the latitude–height distributions of the temperature and static stability in the cloud regions. Because the two satellites have polar (Venus Express) and equatorial (Akatsuki) orbits^7,8^, these radio occultation measurements allow sampling at different latitudes. We used 280 profiles obtained in 2006–2010 by Venus Express and 34 profiles obtained in 2016–2017 by Akatsuki. Figure 1 shows the local times and latitudes of the occultation points for an altitude of 50 km. The profiles were resampled into 1-km-thick bins and then classified into nine latitude bins with a width of 10°. Vertical profiles of the static stability were derived from the obtained temperatures (see Methods for further details). Based on previous *in-situ* measurements^1^, the Venusian atmosphere can be categorised into low-stability (neutrally or weakly stratified; 0–1 K km^−1^), moderately stratified (1–4 K km^−1^), and highly stratified (>4 K km^−1^) layers. Dependencies on local time were not considered, then temperature and static stability distributions were averaged zonally and temporally for the investigation of latitude–height structures.

## Results

### Latitudinal dependence of the mean thermal structure

**Figure 2:**
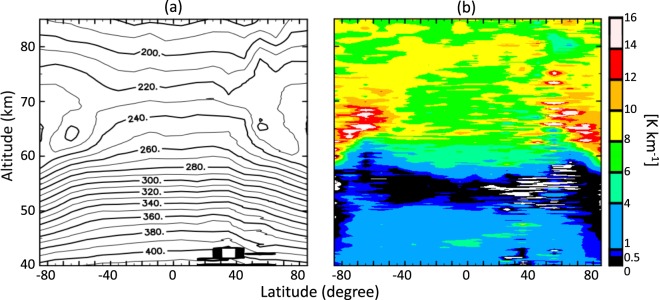
Latitude–height distributions of zonally and temporally averaged (**a**) temperatures and (**b**) static stability obtained from Venus Express and Akatsuki radio occultation measurements.

**Figure 3:**
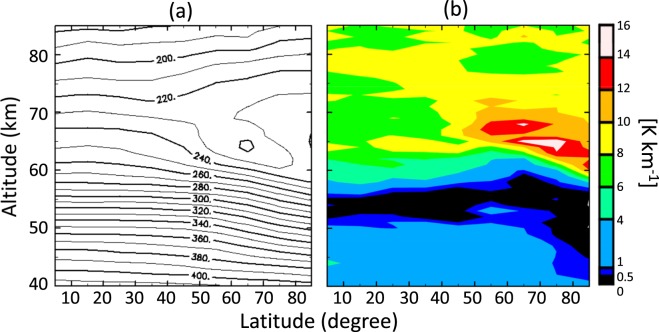
Latitude–height distributions of zonally and temporally averaged (**a**) temperatures and (**b**) static stability obtained from Venus Express and Akatsuki radio occultation measurements. The altitude range is 40–85 km. Data are averaged assuming north–south symmetry to improve latitudinal sampling.

Figure 2a,b respectively shows the latitude–height distributions of the temperature and static stability, which were obtained by averaging all available radio occultation data obtained in Akatsuki and Venus Express missions. The overall structure is hemispherically symmetric; the low stability layer in the lower and middle cloud regions (50–55 km) is relatively thin at low latitude and become thicker at high latitude, and highly stable layers exist at high latitude above 60 km. There were insufficient data around 40°N in and below the cloud layer, and data in the northern and southern cross sections were thus averaged to improve latitudinal coverage and obtain Fig. 3a,b. Hereafter, we discuss the detailed structure on the basis of Fig. 3a,b. Figure 3a shows that the temperature increases with increasing latitude above ~65 km and decreases with increasing latitude below ~65 km, and the cold collar is located at approximately 65° latitude and 65 km altitude. These structures are consistent with previous radio occultation^2–4,9^ and infrared^10,11^ measurements. The static stability is high above ~60 km, especially around the upper half of the cold collar and the lower half of the warm polar regions, which is consistent with the radio occultation measurements of Venus Express^4,12^.

The cloud top height in the infrared region (4–5 and 8.2 µm) changes from 67–68 km at a latitude of <50°–60° to 57–62 km at the pole^13,14^; the trend roughly follows that of the static stability boundary shown in Figs. 2b and 3b. The coincidence might be attributed to radiative cooling at the cloud top, which induces convection below^15^. At lower latitudes, the boundary of static stability is located far below the cloud tops in the infrared and near-infrared region^16^; this might be explained by the stabilizing effect of solar heating in the upper cloud region^15^.

The static stability below an altitude of 60 km changes abruptly around 75° latitude. At lower latitudes, there is a low-stability layer (0–1 K km^−1^) at 50–58 km and a moderately stratified layer (1–4 K km^−1^) below 50 km. The low-stability layer gradually thickens with increasing latitude from the equator to ~70° latitude, and it abruptly expands down to an altitude of ~42 km poleward of 75°, although the static stability is not strictly neutral below an altitude of 50 km as will be shown later.

Such a deep, low-stability layer in the polar sub-cloud region has not been reported previously either for previous radio occultation measurements^4^, which were limited to altitudes above ~45 km, or for *in-situ* measurements^1^, which covered latitudes equatorward of 60°. The present study covers a much wider range of local times and latitudes by retrieving the temperature profiles down to the sub-cloud region globally as shown in Fig. 1. This allows us to show the unique thermal structure of the Venusian atmosphere, namely the layer having low static stability extending down to ~42 km at high latitudes.

### Comparison with VIRA, VIRA-2 and Magellan radio occultation data

**Figure 4:**
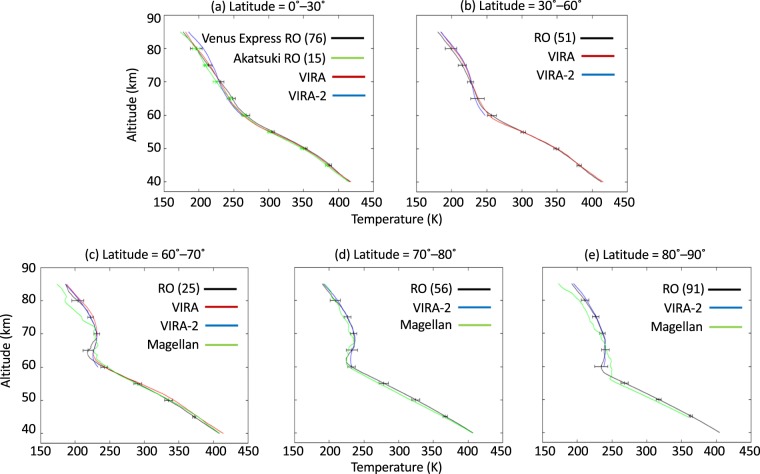
Comparison of the mean temperature obtained from radio occultation measurements (black) with VIRA (red), VIRA-2 (blue) and Magellan radio occultation measurements (green). In each panel, the number within parentheses is the number of radio occultation measurements made at an altitude of 50 km. Latitudinal ranges are (**a**) 0°–30°, (**b**) 30°–60°, (**c**) 60°–70°, (**d**) 70°–80° and (**e**) 80°–90°. The radio occultation data are divided into those obtained on Venus Express (black) and Akatsuki (green) missions at latitudes of 0°–30° (**a**). Error bars in each panel represent the standard deviation.

Figure 4 compares temperature profiles derived from the radio occultation data presently available and those of the empirical models VIRA^17^ (Venus International Reference Atmosphere) and VIRA-2^18,19^ in five latitudinal regions. VIRA-2 profiles are given in terms of the latitude and local time, and we thus compare our results with VIRA-2 results after averaging the VIRA-2 data for local time. The VIRA-2 temperatures below 60 km are considered to be less reliable and omitted because the values at 50–60 km were obtained by interpolating the Venera-15 spectroscopic measurements above 58 km and the VIRA temperatures below 50 km^18,19^. The temperature profiles obtained in the present study agree well with the VIRA data as shown in Fig. 4a–c (see also Fig. S1) while they differ from VIRA-2 data by up to 10 K at latitudes of 0°–30° for altitudes above 70 km (Fig. 4a) and at latitudes of 30°–70° below 70 km (Figs. 4b,c and S1). At latitudes of 70°–90°, which are not covered by VIRA, the temperature difference between the present result and VIRA-2 is as large as 5 K below 65 km, especially around the temperature inversion layer associated with the cold collar (see Figs. 4c–e and S1). Figure 4c–e also include the temperature profiles obtained from Magellan radio occultation measurements. Magellan temperatures at 80°–90° were obtained down to an altitude of 45 km while those at 60°–80° are obtained down to an altitude of 40 km. These temperatures are lower than our measurements except at around altitudes of 57–65 km for latitudes of 80°–90° (see also Fig. S1).

Mean temperature profiles at latitudes of 0°–30° obtained from Venus Express and Akatsuki radio occultation measurements are compared in Fig. 4a. At altitudes of 45–75 km, the observations of Akatsuki are 2–5 K lower than those of Venus Express (see Figs. S1 and S2). The effect of the horizontal drift of the ray path tangential point, which tends to be larger in the Venus Express radio occultation, is negligible (see Supplementary information). A possibility is a long-term variation of the temperature structure. Such a difference is also seen in the comparison between our measurements and the Magellan radio occultation measurements made in 1991 below an altitude of 60 km at high latitudes as mentioned above (Fig. 4c–e). Previous *in-situ* probe measurements are similar in that vertical temperature profiles obtained by Venera 10 in 1975 are 3–12 K warmer than those obtained by Pioneer Venus in 1978, although uncertainties in the Venera temperature data and altitudes are relatively large (4.2 to 8.5 K and ~2 km)^20^.

**Figure 5:**
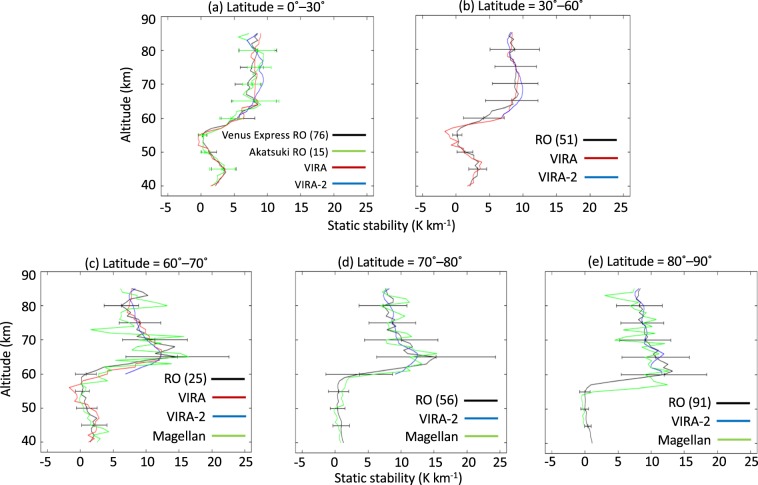
Comparison of the mean static stability obtained from radio occultation measurements (black) with VIRA (red), VIRA-2 (blue) and Magellan radio occultation measurements (green). In each panel, the number within parentheses is the number of radio occultation measurements made at an altitude of 50 km. Latitudinal ranges are (**a**) 0°–30°, (**b**) 30°–60°, (**c**) 60°–70°, (**d**) 70°–80° and (**e**) 80°–90°, and the altitude range is 40–85 km. Only at latitudes of 0°–30°, the radio occultation data are divided into those obtained on Venus Express (black) and Akatsuki (green) missions. Static stability obtained from radio occultation measurements are zonally and temporally averaged. Error bars in each panel represent the standard deviation of the static stability obtained from radio occultation measurements.

Figure 5 shows vertical profiles of the static stability derived from the observed temperatures in latitude bins. These profiles are well consistent with data recorded by VIRA at latitudes of 0°–70° and VIRA-2 at altitudes above 65 km (Fig. 5a–c). At latitudes of 60°–70°, the low-stability layer derived from the Magellan measurements is located at altitudes of 50–60 km, which is well consistent with our result and the results obtained by VIRA (Fig. 5c). At latitudes of 70°–80° and 80°–90°, the low-stability layer derived from Magellan measurements extends down to altitudes of 40 km (Fig. 5d) and 45 km (Fig. 5e), respectively. Although the Magellan measurements at 80°–90° do not seem to reach the bottom of the low-stability layer, the results might support our result that the low-stability layer extends to the sub-cloud level in the polar region as shown in Figs. 2b and 3b. Below an altitude of 60 km, the standard deviation in the low-stability layer is much smaller at latitudes of 80°–90° than in other latitude bins, suggesting an almost steady low-stability layer in the polar sub-cloud level. Standard deviations above an altitude of 60 km at latitudes of 60°–90° larger than those at latitudes of 0°–60° can be attributed to gravity waves excited by convective motions in the low-stability layer, which might be stronger at higher latitudes^12,21^. The mean static stability distributions obtained from the Venus Express and Akatsuki radio occultation measurements are almost consistent with each other (see Fig. S3), although their temperatures differ.

## Discussion

Results of the present study show that below an altitude of 60 km, the low-stability layer in the polar region (>75° latitude) is thicker than that at low latitude and that at middle latitude; this has not been shown by any previous measurements. Previous modelling studies on the vertical convective layer in the Venusian cloud layer^15,22^ suggested that convective motions at altitudes of 50–60 km driven by infrared heating at the cloud bottom are stronger in the polar region than at low and mid-latitudes because the infrared cooling of the upper cloud is more effective at higher latitudes. The strong convective motions might penetrate the underlying stably stratified layer^23^, and the convection layer thus might be thicker at higher latitudes. Enhancement of convection by radiative cooling at upper levels is thought to occur also in Martian night-time ice clouds^24^. However, the change in the Venusian convective layer depth along the latitude seems to be too small to account for the latitudinal distribution of the static stability below an altitude of 50 km shown in Fig. 3b in terms of the latitudinal difference in solar heating and/or convective motions.

Another possibility is that the thermal structure below an altitude of 60 km might be affected by the mean meridional circulation and/or wave activity. It has been suggested that the mean meridional circulation in and below the cloud layer is complex^5,25^. If there is a strong downward flow associated with the mean meridional circulation below the polar cloud layer, it would induce adiabatic heating and destabilize the polar atmosphere, although the structure of the mean meridional circulation in and below the cloud layer is unknown. It has also been suggested that non-axisymmetric eddies generated by baroclinic instability induce a non-axisymmetric temperature disturbance in the polar vortex^26–28^. Recent numerical studies showed that eddies with various periods and spatial scales might appear in the cloud layer^28–30^, and that Rossby-like waves generated by baroclinic instability produce planetary-scale cloud features in the low-stability layer^31^. The unique thermal structure presented in this study might result from the meridional heat transport induced by such disturbances. The atmospheric dynamics at these levels could be constrained by investigating the heat budget associated with the thermal structure.

## Methods

In the radio occultation experiment, the spacecraft orbiting Venus transmits radio waves to the tracking station on the Earth as it passes behind the atmosphere as seen from the tracking station. The received signal is recorded by the tracking station, and the frequency time series is retrieved offline. Using an ephemeris, the Doppler shift due to the motion of the spacecraft and the ground receiver is removed to obtain the frequency residual generated by the atmosphere. This residual is used to obtain the atmospheric structure using an Abel transform to get the refractivity profile, which is converted to a density profile using an assumed atmospheric composition. The hydrostatic equilibrium and equation of state are then used to get temperature and pressure profiles. In converting the radio occultation data into a temperature profile, the same equation of state, the same atmospheric composition and the same upper boundary condition are used for Venus Express and Akatsuki spacecraft.

Two types of recording system are employed in a radio occultation experiment: closed-loop and open-loop systems. The former systems obtain the real-time frequency and amplitude using phase-lock loop technology. The latter systems heterodyne the received signal with a signal from a local oscillator to produce a signal of lower frequency, which is digitised for offline processing. The radio occultation data of Venus Express were recorded using both types of system, whereas only an open-loop system was employed for the data of Akatsuki. The open-loop data allowed the retrieval of temperature profiles down to an altitude of approximately 40 km. Using the closed-loop data, the bottom altitude of the retrieved temperature profile was limited to 45–50 km^4,32^. The closed-loop data are not suitable for investigating the sub-cloud region, and we thus used the X-band open-loop data obtained on Venus Express and Akatsuki missions in the present study. Overviews of the Venus Express and Akatsuki radio occultation measurements are presented elsewhere^4,32–35^.

The error analysis of the Venus Express radio occultation is based on the linear error propagation method proposed by Lipa and Tyler^36^ and explained by Tellmann *et al*.^4,12^. The uncertainties in temperature and pressure are typically less than 1 K and 0.1 percent, respectively. The error in Akatsuki radio occultation measurements was evaluated by simulating with a forward calculation the radio frequency perturbation generated by a localized temperature perturbation and by comparing the frequency perturbation with the noise level of the frequency time series. The error is of the order of 0.1 K; details were given by Imamura *et al*.^33,34^. The above errors are random statistical errors; other sources of error include uncertainty in the spacecraft position, deviation of the atmosphere from spherical symmetry, and a possible multipath effect. Errors due these sources have not yet been estimated in detail.

We obtained the static stability from the temperature distributions using the formula

1 $$S=\frac{dT}{dz}+\frac{g}{{C}_{p}},$$

where *T* is the temperature, *z* is the altitude, *g* is gravitational acceleration, and *C*_*p*_ is the specific heat at constant pressure. Both *g* and *C*_*p*_ vary with altitude, and the altitude dependence of *C*_*p*_ is based on the work of Seiff *et al*.^17^. Recently, the adiabatic lapse rate calculated with consideration of a mixture of two gases, 96.5% CO_2_ and 3.5% N_2_, was presumed to give variable abundances with altitude^37^. Nevertheless, the difference between the adiabatic lapse rate obtained for the two-gas mixture and the VIRA values is less than 0.02 K km^−1^, which is much smaller than the variance in the static stability shown in Fig. 5.

## Supplementary information

Supplementary Information.
