# Supplementary material for: Thermal structure of the Venusian atmosphere from the sub-cloud region to the mesosphere as observed by radio occultation
Source: Sci Rep. 2020 Feb 26;10:3448. doi: 10.1038/s41598-020-59278-8 (PMC7044293; doi:10.1038/s41598-020-59278-8)
Supplement: Supplementary file 1 — Supplementary Information. [file 41598_2020_59278_MOESM1_ESM.pdf]

# Supplementary information

# Thermal structure of the Venusian atmosphere from the sub-cloud region to the mesosphere as observed by radio occultation

*Hiroki Ando[1] (hando@cc.kyoto-su.ac.jp), Takeshi Imamura[2], Silvia Tellmann[3], Martin Pätzold[3], Bernd Häusler[4], Norihiko Sugimoto[5], Masahiro Takagi[1], Hideo Sagawa[1], Sanjay Limaye[6], Yoshihisa Matsuda[7], Rajkumar Choudhary[8] and Maria Antonita[9]

1: Kyoto Sangyo University, 2: The University of Tokyo, 3: Universität zu Köln, 4: Universität der Bundeswehr München, 5: Keio University, 6: Keio University, 7: Tokyo Gakugei University, 8: Vikram Sarabhai Space Center, 9: Indian Space Research Organization

**1. Detail of the comparison of the temperature distributions obtained by the radio occultation measurements, VIRA and VIRA-2**

Fig. S1 shows temperature differences among the radio occultation measurements, VIRA and VIRA-2. Fig. S2 shows the temperature distributions obtained from the Akatsuki and Venus Express radio occultation measurements. Although the temperature obtained on the Akatsuki mission is 2–5 K lower than that obtained on the Venus Express mission at all altitudes (see also Fig. S1), the shape and curvature of these temperature profiles are similar. The static stabilities obtained from these radio occultation measurements are then almost consistent as shown in Fig. S3.

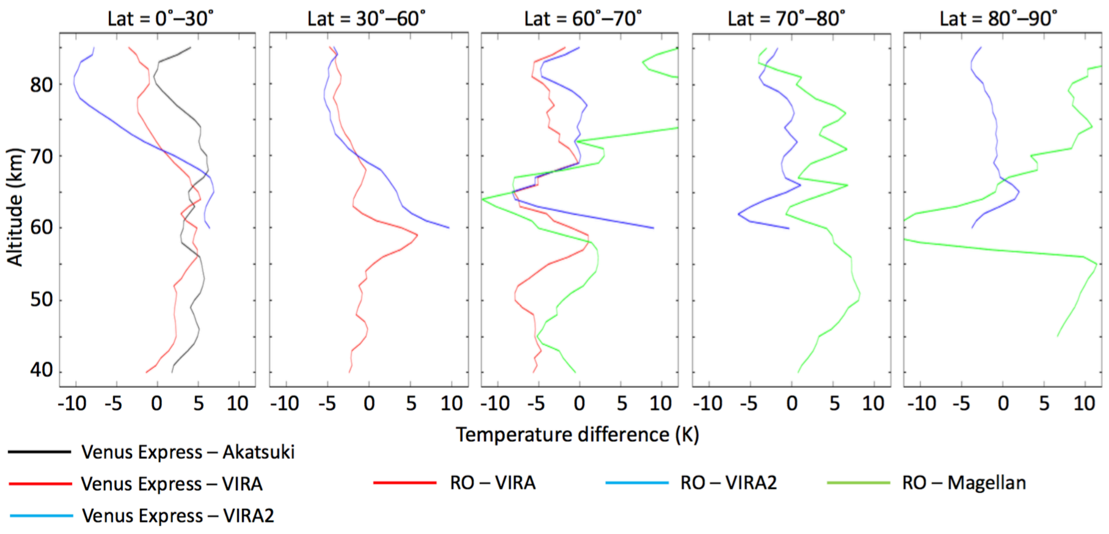


**Figure S1 | Differences in temperature among radio occultation measurements,**

**VIRA and VIRA-2.** At latitudes of 0°–30°, the temperature differences between Venus Express and Akatsuki (black), between Venus Express and VIRA (red), and between Venus Express and VIRA-2 (blue) are shown. At other latitudes, the temperature differences between the present study and VIRA (red) and between VIRA-2 (blue) and Magellan radio occultation measurements (green) are shown.

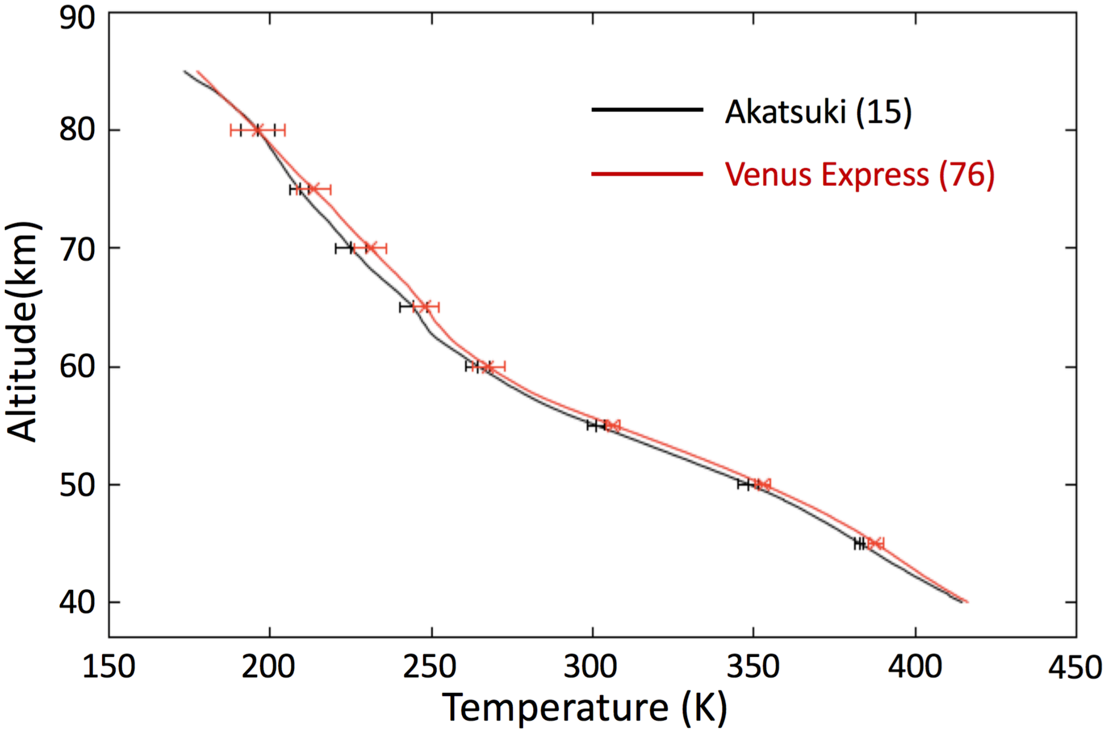


**Figure S2 | Comparison of mean temperatures at altitudes of 40–85 km for latitudes of 0°–30° obtained from radio occultation measurements made on (black) Akatsuki and (red) Venus Express missions.** The number within parentheses in each

panel is the number of radio occultation measurements made at an altitude of 50 km. An error bar represents the standard deviation of the temperature.

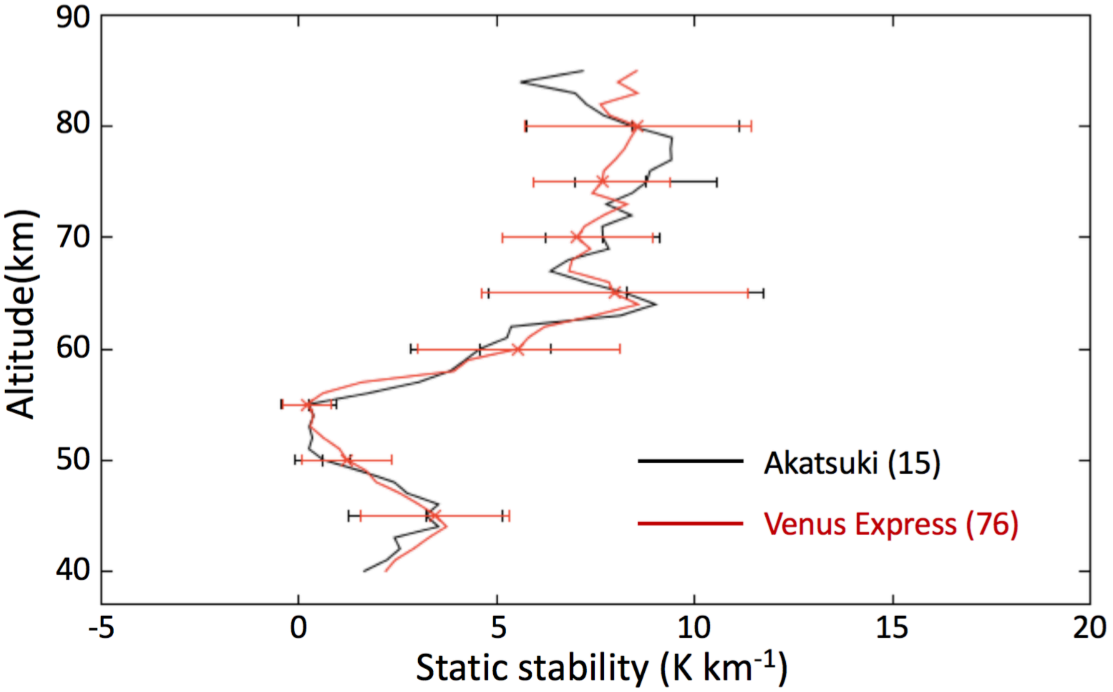


**Figure S3 | Comparison of mean static stabilities at altitudes of 40–85 km for latitudes of 0°–30° obtained from radio occultation measurements made on (black) Akatsuki and (red) Venus Express missions.** The number within parentheses in each panel is the number of radio occultation data measurements made at an altitude of 50 km. An error bar represents the standard deviation of the temperature.

**2. Measurement error due to horizontal drift**

Venus Express had a polar orbit. Horizontal drift of the ray path tangent point thus generated measurement error in the temperature at low latitudes. The error is estimated here following the work of Kursinski et al. (1997).

The bending angle error, $\delta\alpha(z, z_0, y)$, due to horizontal drift of the ray path tangent point is expressed to the first order as

$$\delta\alpha(z, z_0, y) = \Delta y(z, z_0, y)\frac{\partial\alpha}{\partial y}(z, y) \ , \qquad \text{(A1)}$$

where $y$ represents latitude and $z$ is the height of the error, $z_0$ is the altitude of the lower integration limit of the Abel transform and the altitude of the refractivity retrieval, and $\Delta y$ is the horizontal distance that the ray path tangent point drifts between $z$ and $z_0$. For simplicity, we assume that $\alpha$ is proportional to the vertical density gradient such that $\alpha \sim c\rho/H$, where $c$ is a scale factor, $\rho$ is the mass density and $H = RT/g$ is the density scale height, with $R$, $T$ and $g$ respectively being the gas constant, temperature and gravity acceleration. Making this assumption, it follows that

$$\frac{1}{\alpha}\frac{\partial\alpha}{\partial y}(z, y) \sim \frac{1}{\rho}\frac{\partial\rho}{\partial y}(z, y) - \frac{1}{T}\frac{\partial T}{\partial y}(z, y) \ . \qquad \text{(A2)}$$

In the case of Venus, the cyclostrophic balance can be applied as

$$\frac{u_c^2 tan\varphi}{a} = -\frac{1}{\rho}\frac{\partial p}{\partial y} , \quad \text{(A3)}$$

where $a$ is the radius of Venus, $u_C$ is the cyclostrophic wind, $p$ is pressure and $\varphi$ is the latitude. Taking the vertical gradient of (A3) gives

$$\frac{\partial}{\partial z}\left(\frac{\rho u_c^2}{a}\right) = -\frac{1}{tan\varphi}\frac{\partial}{\partial y}\left(\frac{\partial p}{\partial z}\right) = \frac{g}{tan\varphi}\frac{\partial \rho}{\partial y} . \quad \text{(A4)}$$

The first term on the right-hand side of (A2) can therefore be rewritten as

$$\frac{1}{\rho}\frac{\partial \rho}{\partial y}(z,y) = \frac{tan\varphi}{ag}\left(2u_c\frac{\partial u_c}{\partial z} + \frac{u_c^2}{\rho}\frac{\partial \rho}{\partial z}\right) = \frac{tan\varphi}{ag}\left(2u_c\frac{\partial u_c}{\partial z} - \frac{u_c^2}{H}\right). \quad \text{(A5)}$$

The second term on the right-hand side of (A2) is related to the vertical gradient of the cyclostrophic wind such that

$$\frac{\partial}{\partial z}\left(\frac{u_c^2 tan\varphi}{a}\right) = -\frac{R}{H}\frac{\partial T}{\partial y} = -\frac{g}{T}\frac{\partial T}{\partial y} . \quad \text{(A6)}$$

Accordingly, (A2) is finally described as

$$\frac{1}{\alpha}\frac{\partial \alpha}{\partial y}(z,y) = \frac{tan\varphi}{ag}\left(4u_c\frac{\partial u_c}{\partial z} - \frac{u_c^2}{H}\right). \quad \text{(A7)}$$

The fractional error in the bending angle due to horizontal drift of the ray path tangent point is then written as

$$\frac{\delta\alpha}{\alpha} = \frac{tan\varphi}{ag}\left(4u_c\frac{\partial u_c}{\partial z} - \frac{u_c^2}{H}\right)\Delta y . \quad \text{(A8)}$$

The radius ($a$) is 6050 km, gravitational acceleration ($g$) is 8.87 m s$^{-2}$, the density scale height ($H$) is fixed at 5 km and the latitude ($\varphi$) is fixed at 20°. We consider the two

altitude ranges of 40–50 km and 60–70 km. In the case of the Venus Express radio occultation measurements made at low latitude, $\Delta y$ is approximately $10^3$ km at altitudes of 40–50 km and $10^2$ km at altitudes of 60–70 km, which corresponds to latitudinal movement of ~10° and ~1°, respectively. Inserting these values into (A8) to calculate the temperature measurement error ($\delta T$) yields $\delta\alpha/\alpha \sim 6.7 \times 10^{-4}$ and $\delta T \sim 0.05$ K at altitudes of 40–50 km and $\delta\alpha/\alpha \sim 2.7 \times 10^{-4}$ and $\delta T \sim 0.03$ K at altitudes of 60–70 km. These $\delta T$ values are smaller than the measurement error of 0.1 K, which is associated with the stability of the oscillator onboard the spacecraft.

## References

Kursinski, E. R., Hajj, G. A., Schofield, J. T., Linfield, R. P. & Hardy, K. R. Observing Earth's atmosphere with radio occultation measurements using the Global Positioning System. *J. Geophys Res*. **102**, 23429–23466 (1997).

## Figure legends

**Figure S1 | Differences in temperature among radio occultation measurements,**

**VIRA and VIRA-2.** At latitudes of 0°–30°, the temperature differences between Venus Express and Akatsuki (black), between Venus Express and VIRA (red), and between Venus Express and VIRA-2 (blue) are shown. At other latitudes, the temperature differences between the present study and VIRA (red) and between VIRA-2 (blue) and Magellan radio occultation measurements (green) are shown.

**Figure S2 | Comparison of mean temperatures at altitudes of 40–85 km for latitudes of 0°–30° obtained from radio occultation measurements made on (black) Akatsuki and (red) Venus Express missions.** The number within parentheses in each panel is the number of radio occultation measurements made at an altitude of 50 km. An error bar represents the standard deviation of the temperature.

**Figure S3 | Comparison of mean static stabilities at altitudes of 40–85 km for latitudes of 0°–30° obtained from radio occultation measurements made on (black) Akatsuki and (red) Venus Express missions.** The number within parentheses in each panel is the number of radio occultation data measurements made at an altitude of 50

km. An error bar represents the standard deviation of the temperature.
